# Decoding Investor Sentiments in the Indian Stock Market: A Structural Equation Modelling Approach

**DOI:** 10.12688/f1000research.156635.2

**Published:** 2025-05-02

**Authors:** Arvind Nain, N.S. Borha, Farman Ali, Anu Sayal, Pradeep Suri, Sanjay Singh Chauhan, Vasim Ahmad

**Affiliations:** 1 Graphic Era (Deemed to be University), Uttaranchal University, Dehradun, Uttarakhand, India; 2 Management, Uttaranchal University, Dehradun, Uttarakhand, 248007, India; 3 Taylor’s Business School, Taylor’s University, Malaysia, Subang Jaya, 47500, Malaysia

**Keywords:** Investment decision, Investors sentiments, stock market, behavioural finance, irrationality of Investor

## Abstract

**Background of the study:**

This research examines how psychological and social biases affect individual investors’ investing decisions. Investor sentiment significantly influences financial markets, frequently causing stock prices to deviate from their intrinsic values. In rising economies such as India, where retail investors are significantly affected by psychological factors, comprehending these attitudes is crucial.

**Methods:**

This study analyses data from a comprehensive questionnaire that was conducted throughout the nation and included 552 retail investors. The investigation employed structural equation modelling (SEM) to identify the elements that influence the decision-making of individuals who invest in the Indian stock market.

**Findings:**

The research offers insight on the influence that investor attitude has on investment decision-making as well as the factors that precede it. The study demonstrates that investors make financial decisions based on sentiment. In addition to assessing the efficacy of the Indian financial market, this study sought to ascertain the rationality of investors’ choices by exploring the factors that influence their decision-making process.

**Conclusion:**

The outcome of the study shows that information seeking, anchoring, herding, representativeness, and overconfidence all have a big impact on investors. Moreover, the study has proven investors’ irrationality and stock market inefficiency. The findings may be employed to further examine the trading practices of international investors and encourage further study in the field of behavioural finance.

## Introduction

Over the last decade, academics have focused on the relationship between investor emotions and market performance. Investor sentiment refers to the anticipated future earnings (returns) and the associated risk that market participants have regarding their investments ( De Long *et al., *1990). Traditional hypotheses in the stock market primarily emphasized the efficient market hypothesis (EMH) and random walk theory, neglecting the importance of investor mood ( Vishwanath & Krishnamurti, 2009). Nevertheless, they were unable to elucidate the diverse behaviours exhibited by investors in the capital market. Investor sentiment significantly influences the capital market, leading to fluctuations in stock prices and creating uncertainty over future returns. Over the past several decades, the financial environment in India has seen substantial transformations, transitioning from an economy focused on savings to one that emphasizes investment. The ninth goal of the Sustainable Development Agenda strives to construct resilient infrastructure, encourage inclusive and sustainable industrialization, and encourage innovation. To achieve this, we must support innovative, sustainable technology and enable widespread, equal access to information and financial markets. Research has shown that individual investors often make irrational decisions, with factors such as heuristics, mental accounting, greed, fear, mental dissonance, and anchoring affecting decision-making ( Schmeling, 2009; Kumari & Mahakud, 2016; Narasimha & Mushinada, 2020). Herding behavior in the Chinese and Indian stock markets is more prevalent amidst market upswings, while in India, it occurs during market fluctuations ( Wen *et al*., 2019). Factors such as risk tolerance, the soundness of the Indian economy, media concentration on the stock market, political stability, and government policy towards business significantly affect retail investors’ attitudes regarding equity stock investment. Financial literacy is also a key factor in individual saving, with older individuals being more likely to have positive savings. Classical financial theory presupposes a well-functioning market, rational investors, and asset prices that accurately reflect the most important market information ( Ouzan, 2020). Because the present value of future cash flows defines asset values in an efficient market, asset prices are not believed to be impacted by investor mood. Nevertheless, there are many examples in financial market history when classical theory fails to adequately explain the actions of asset values, particularly during times of speculation and crises. Finding out how individual, foreign, and institutional investors affect the performance of the stock market is the main goal of this article. We delve further into the topic of how investors act and its impact on the Indian stock market.

Researcher ( Al-Tamimi, 2006) found that an investor’s investing attitude, associated norms, and apparent behavioural control influence their investing intent. Gender plays a significant role in the relationships between investment attitude and psychological components, behavioural intentions and attitude, behavioural intention and subjective standards, and behavioural intention and perceived behavioural control. Dash (2010) examined the key characteristics driving investment behaviour and how these aspects affect investors’ risk tolerance and technique process among people of various ages. De *et* al. (2011) found that behavioural biases had a greater economic impact on individual investors than other investors, and overconfidence caused more wealth loss than disposition effects. Chaudhary (2013) explored the meaning and significance of behavioural finance and its applicability in investment decisions, revealing that anchoring, overconfidence, herd behaviour, over and under-reactions, and loss aversion are all factors that influence investors’ investing decisions. Kartašova (2013) investigated the characteristics that shape irrational individual investor behavior in the Lithuanian stock market, using literature review, theoretical insight comparison, networking, benchmarking analogy, and generalization. He found that overoptimism, anchoring, mental accounting, and herd actions had a higher impact on their financial decision-making process. Kumari and Mahakud (2015) experimentally examined investor sentiment and stock return volatility in the Indian stock market, finding that volatility persists and has clustering, asymmetry, and leverage effects due to investors’ psychological biases and herding tendencies. Thulasipriya (2015) investigated investors’ preferences for various investment channels and their investing pattern, finding that Indian investors, even high-income, well-educated, paid, and self-sufficient, prefer to play it safe. Lad and Tailor (2016) analysed the stock exchanges in India, finding that ordinary stock investors were unable to capitalize on market development and volatility. Prasad *et al.* (2017) examined concerning the psychological impact of disposition and overconfidence was identified in the Indian equity market.

Sarkar and Sahu (2018) investigated the impact of demographic factors, awareness, and perceived risk attitude on investment behaviours, finding that demographic characteristics, knowledge, and risk perception have a considerable impact on individual stock market investors’ investment behavior. There is a school of thought within behavioural finance theory that maintains investors act irrationally when drawing speculative conclusions ( Shantha, 2019). Prospects including risk aversion, loss aversion, mental accounting, and crowding behaviour, as well as heuristics including attachment, gamblers’ misinterpretation bias, and competence bias, inform the resolution generation approach. Ordinarily, private investors acquire stocks during periods of upward trends and divest them during periods of declining trends.

Some factors that have a detrimental impact on the stock market include war crimes, fraud, government instability, attacks, scams, high oil prices, and instability on a global and domestic scale. Accordingly, we choose to investigate the aforementioned connection between psychological and sociological factors. The impact of investors’ irrational, rational, and decision-making behaviours on their perceptions of stock index and market movements is the focus of this research. The research article is divided into several main components. It starts with a background and literature review, which provides context and highlights key studies. The technique part describes the research procedure, which is then followed by the hypothesis formulation. The study subsequently explores the data’s reliability and validity in order to assure its accuracy. The data analysis section describes the procedures used to analyze the results. The results are finally evaluated, leading to a conclusion that summarizes the study’s principal findings. Finally, recommendations are made based on the research findings.

## Background of the study: Literature review

Classical finance, which is founded upon rational expectations rather than investor sentiment, disregards investor sentiment ( Friedman, 1953). The asset pricing model assumes that future cash flows and discount rates determine asset returns. It seems logical that rational arbitrage brings asset values closer to their fundamentals. Smales (2014) examined collective news perception and implied volatility index swings. The implied volatility index negatively correlates with news sentiment. The implied volatility index fluctuates more after unfavourable news. Garg and Varshney (2015) investigated the momentum impact in the Indian equity market from May 2000 to April 2013 utilising significant CNX 500 businesses in four sectors: automobile, banking, pharmaceutical, and Information technology. They found a momentum impact in the Indian stock market, supporting behavioural hypothesis, traders’ underreaction and overreaction, to firm-specific news are other causes of momentum ( Daniel *et al.*, 1998; Hong *et al., *2000).

An early inaction and subsequent overreacting to firm-specific news as well as investors’ overconfidence in their capabilities, generate momentum in the stock market ( Chan *et al.*, 1996; Hong & Stein, 1999; Hirshleifer, 2001). Market information flow affects asymmetric volatility in four rising market economies: Brazil, Russia, India, and China ( Naik & Padhi, 2015). All four nations have statistically significant asymmetric equity return volatility during the research period. Due to a “leverage effect,” negative shocks induce higher market volatility than positive shocks of the same magnitude in these nations. Kumari and Mahakud (2015) experimentally examined investor sentiment and stock return volatility in the Indian stock market. Volatility persists and has clustering, asymmetry, and leverage effects due to investors’ psychological biases and herding tendencies. Thulasipriya (2015) investigated investors’ preferences for various investment channels as well as their investing pattern *.*

Lad and Tailor (2016) examined how India’s stock markets have changed structure, ups and downs, and benchmarks, making them functional. Ordinary stock investors, however, were unable to profit from the market’s growth and volatility. The validity findings showed that investor investment decisions are influenced by behavioural biases. Aruna and Rajashekar (2016) examined the many factors that influence the investment decisions of retail investors. The study indicated that these traits are subject to change throughout time. Researchers concluded that gold outperforms other alternative investments based on a comprehensive analysis of daily gold prices, daily stock index (Nifty50), and daily bond (India Govt.) yield from 2012-2019. Gold investing is safer than stock and bond investment because gold prices have less daily fluctuation ( C.V, 2017). An empirical study aimed at identifying differences in investing behavior preference between individual investors in Mainland China and Hong Kong ( Mak & Ip, 2017).

Prosad *et al. *(2017) revealed substantial experimental evidence of the disposition consequence and overconfidence in the Indian equity market between 2006 and 2013. Sarkar and Sahu (2018) tested how demographics, awareness, and risk attitude affect investment behaviour. The study found that demographics, expertise, and risk perception influence stock market investors’ behavior. Empirical evidence substantiated the overconfidence hypothesis. Mushinada and Veluri (2018) overconfident investors respond too strongly to confidential information and too softly to disclosures. EGARCH found that when predictions come true, self-attribution bias increases investor confidence and trade volume. Ultimately, the excessive confidence of investors leads to a substantial increase in the volatility of returns due to their active trading. Beer *et al. *(2018) looked at the possibility that investor insolence impacts European accruals anomalies. The author discovered a correlation between investor psychological state and the mispricing of accruals in European markets. A greater impact was felt on stocks with arbitrary, difficult-to-arbitrage prices. Vidya and Satheesh (2019) examined a variety of behavioural biases, including heuristic bias, optimistic bias, herd biases, loss aversion bias, and overconfidence bias, in order to investigate any relationships between demographic variables and investor behavioural biases. Areiqat *et al. *(2019) examined the impact of a number of significant behavioural finance variables covered in the financial literature (overconfidence, loss aversion, risk perception, and herding) that may influence the stock investing decision-making at Amman Stock Exchange (ASE), as well as the relative relevance of these variables. Narasimha and Mushinada (2020) proposed that overconfidence displayed the greatest level of relative importance. The researcher further analysed self-attribution bias, overconfidence bias, and investors’ ability to adjust to market fluctuations. Nevertheless, investors sporadically exhibited behavior that seemed ridiculous.

Cui and Zhang (2020) examined investor emotion and stock market risk of catastrophe. High-feeling organizations were shown to be more vulnerable to stock price declines. The crash risk was more significantly influenced by investor mood for enterprises that had higher debt ratios, default risks, and analyst forecast dispersion. According to Ye *et al.* (2020) herding behavior is prevalent in China’s A-share market, especially during periods of extreme bear market conditions, but not during periods of mild bull market conditions. This supports the study hypothesis, which holds that herding behavior is a result of loss aversion and consequential anticipation.

Bouteska and Regaieg (2020) investigated the impact of loss aversion and overconfidence on the performance of enterprises in the United States. They initially examined the effects of loss aversion on the economic performance of these organizations. Dhall and Singh (2020) analysed the collective behavior of investors in the business by utilizing NSE data to study herding mentality. The author analysed the phenomenon of herding behavior prior to, during, and following the COVID-19 pandemic. Maghyereh and Abdoh (2022) identified a differentiation between the Global Financial Crisis (GFC) and the COVID-19 crisis in terms of how sentiment information influenced market volatility. Scientists investigate the predictive capacity of emotions during crises as they might reduce ambiguity on the actual fluctuation of assets. The discovery demonstrated that emotion is a more accurate predictor of realized volatility during periods of crisis, contingent upon the kind of asset. Cevik *et al. *(2022) investigated the relationship between optimistic and pessimistic investor perspectives and stock market returns and volatility in the Group of 20 nations. The Google Search Volume Index was utilized to assess investor opinion toward COVID-19 and its vaccination. During the period from March 2020 to May 2021, the author discovered significant correlations between positive and negative investor sentiments, stock market returns, and market volatility. Specifically, the returns on stocks increase when investors exhibit optimism and decrease when they display pessimism, particularly at lower quantiles. Optimistic investor sentiment decreases market volatility, whereas pessimistic investor sentiment increases market volatility. Several investigations Choudhary and Singhal (2020); S. Kumar *et al.* (2021); Sahoo and Kumar (2021) and Kartal *et al.* (2022) have identified the causal connections between the stock market and the stock market crises. Previous studies He *et al. *(2020) and Stawiarski (2021) have established a causal connection between India and Asian nations. The GARCH model applied Narasimha and Mushinada (2020) to analyse the sentiments of the investors. It revealed that the Indian stock market exhibited higher levels of volatility compared to industrialized nations throughout the crisis. Recent developments in behavioural economics have revealed the presence of volatility clustering in the Indian stock market. This has been achieved by analysing time series data and employing predictive modelling techniques to understand investor behaviour. Experimental results by Khaing *et al.* (2020) revealed that trend extraction with additional criteria of news effect curve on stock extracted more trends during the crisis. In addition to new factors, the stock trend extraction conclusions are more consistent with actual stock price movement. Chen (2021) constructed an innovative neural network model to enhance the forecasting capabilities and emotional analysis of investors. Belciug *et al.* (2021) tested a quantitative and statistical concept based on a competitive/collaborative method to predict a prospective stock market transaction price for a security while an economic upsurge.

Biswas *et al.* (2021) analysed the models and techniques employed for predicting stock market price fluctuations. They focused on the advantages and disadvantages of these approaches, particularly in the context of a crisis. Debata *et al.* (2018) identified a positive (negative) correlation between investor sentiment and liquidity (illiquidity). Results also revealed that the sentiment of international investors has a major impact on the liquidity of emerging stock markets. Cheema *et al.* (2020) discovered a favourable correlation between emotion throughout the bubble phase and market returns, which becomes unimportant once the bubble bursts. Investors view increased indebtedness in the energy sector and higher operating income in the telecom sector as predictors of future economic instability and worsening the condition, according to the results of a partial least squares regression model for consumer confidence. On the other hand, there was a positive relationship between the stock index and consumer confidence ( Zorio-Grima & Merello, 2020). Investors are emotionally susceptible. Due to Australia’s status as a resource-rich but capital-poor nation, this article analyses and evaluates the influence of investor mood on performance returns. Consumer Confidence Index (CCI) and trade volume are sentiment proxies used to examine aggregate, cross-sectional, and predictive regression impacts while controlling for macroeconomic variables ( Banchit *et al.*, 2020). Haroon and Rizvi (2020) examined the association between the mood of investors as influenced by COVID-19-related media reports and the volatility of stock markets. According to the conclusions of the study, the rise in uncertainty and volatility on the financial and stock markets was a result of news related to COVID-19. Ambros *et al.* (2021) analysed the influence of COVID-19-related news on eight distinct stock market indices. Even if stock returns were unaffected through changes in the dimensions of COVID-19-related news, their empirical findings indicate that the volatility of European stock markets rose dramatically as a result of the increase in COVID-19-related news coverage. Iyke and Ho (2021) analysed Google search phrases pertaining to COVID-19 to gauge investor interest, and then analysed the association between investor attention and stock market indexes in 14 African nations. Researchers concluded that investor attention is one of the most influential determinants of stock returns, and that a rise in investor attention is associated with a fall in stock returns in Botswana, Nigeria, and Zambia. In both Ghana and Tanzania, investor sentiment and the returns on stock investments exhibit a favourable link. Narayan *et al.* (2021) developed six distinct global sentiment indicators for COVID-19 based on a word search of 45 major media pieces. These variables include COVID, medical, vaccine, travel, ambiguity, and collective sentiment. These signs were useful for analysing the effects of the global pandemic, according to the conclusions of the research. Prior to 2012, the unpredictability of economic policy had a clear and provable adverse impact not just on investor sentiment but also on the short-term stability of the financial system, with the impact on investor sentiment being far bigger than that on the financial system. These impacts began to come to the forefront in 2008, which was right in the thick of the global financial crisis. Additionally, investor optimism had a favourable and steadily expanding influence on financial stability up to the year 2010, after which the beneficial effect gradually vanished ( Qi *et al., *2022). The researcher found that style returns have predictive power for future stock returns during moments of elevated sentiment, but not during periods of low sentiment ( Ashour *et al.*, 2022). Sakariyahu *et al.* (2023) stated that the volatility of the sampled indexes at any one time is significantly influenced through the sort of news (good/bad), extreme occurrences, and, more crucially, investor sentiment.

Li and Xing (2023) studied the relationship between sentiment and synchronicity in the Chinese stock market, discovered that investor sentiment had a considerable positive effect on the synchronisation of stock returns. Liu *et al.* (2024) demonstrated that coal price fluctuations have a considerable negative influence on Chinese stock market returns, but the resultant degree varies over time, notably amid recessions. According to research, investor psychology impacts the capital market’s performance and the manner in which investors interpret basic data, which in turn impacts the correlation between these two variables and stock returns ( Fonou-dombeu *et al.*, 2024). Researchers ( Gao *et al.*, 2024) indicates that both trading and online emotions have a detrimental impression on stock returns through the mediating result of liquidity. The results of a comparison between the partial least squares (PLS)-based investor sentiment index, three linked sentiment metrics from prior research, and six separate sentiment proxies showed that performed the best in predicting stock volatility on the Chinese stock market ( Song *et al., *2023). There is a negative contemporaneous link between investor sentiment and market return, according to research on the function of investors in the stock market ( Phan *et al*., 2023). Ayinuola and Adetiloye (2023) discovered that the sentiment index is adversely and strongly related to stock return volatility. Bashir *et al.* (2024) examined that stock price crashes are more likely when market mood rises, and that this effect is exacerbated through investors’ herd mentality. Ivanov and Kobets (2025) investigated a statistically significant positive relationship between news sentiment and the S&P 500 market index. During times of market volatility, stock prices react much more to news. Dsouza *et al*. (2025) found that strong ESG initiatives improve market value and operational effectiveness, providing valuable insights for policymakers, corporate leaders, and investors. Classical finance is based on rational expectations, with asset prices defined by future cash flows and discount rates. However, multiple studies have shown that investor mood has a major impact on market volatility, asset mispricing, and return anomalies. To gain a more complete understanding of market dynamics, it is necessary to bridge the gap between classical asset pricing theories and behavioural finance insights.

Investigating investor behaviour through the lens of a contemporary social and psychological paradigm can provide a fresh perspective on financial markets. A study combining classical and behavioural finance theories and testing their impact on stock market indices would address this research gap. The significance of social and psychological factors in affecting investor decision-making, particularly in varied market scenarios (e.g., financial crises, economic recovery), is understudied. Understanding how these elements influence investor reactions to news and economic shocks may help predictive models of market behaviours.

## Methods

Individual investors who invested in the Indian stock market through stock brokerage firms in India comprise the study’s population. All of the investors are drawn from various cities with a high concentration of retail investors. When the population being sampled is exceptionally large, it becomes extremely difficult to design a sampling frame. Even though the population of India’s individual investors who invest in equity shares is extremely large and difficult to identify, it is challenging to design a sample frame for this group. Nevertheless, we developed a sample frame based on data from the Stock Holding Corporation of India Ltd. The investigated population was chosen arbitrarily in advance based on trading and consultation with professionals from Stock Holding Corporation of India Ltd. Samples were collected using stratified random sampling. Stratified random sampling is a form of confined probability sampling in which the population has been split into strata based on their homogeneity, and observations are randomly selected from all of the strata. The response rate of investors was 81.5%, which means that out of the 800 online surveys that were mailed out, only 571 were filled out and answered. Out of 571 accurately filled up questionnaires, 552 were utilised for analysis. Geographic location was the most relevant criterion for stratifying the whole universe, according to the study. Based on this, the geographical structure of India may be separated into five subregions: the northern area, the eastern region, the central region, the western region, and the southern region. The questionnaire’s final objective is to analyse the behaviour of retail investors. In this section, data were gathered using a 5-point Likert scale, where “1” indicates Strongly Disagree, “2” indicates Disagree, “3” indicates Neutral, and “4” indicates Agree. “5” indicates Strong Agree. Cronbach’s Alpha is the most common and extensively used reliability measure. Structural Equation Modelling (SEM) is employed for studying this set of measurements because of its capacity to evaluate complex correlations between observable and latent variables while accounting for measurement errors ( Hair *et al.,* 2019). Given that the study’s goal is to analyse the behaviour of retail investors in India utilizing multi-item constructs measured on a 5-point Likert scale, SEM provides a strong framework for investigating both direct and indirect correlations between variables ( Byrne, 2016). Furthermore, the study uses stratified random sampling to collect data from several geographic regions, necessitating a method that can account for structural differences among strata. SEM provides a comprehensive framework for comprehending complex interdependencies that cannot be successfully captured using simpler statistical techniques such as multiple regression or factor analysis.

## Hypothesis

H0:
There is no association between factors (behavioural biases variables) and investors decision making.

H1:
There is an association between factors (behavioural biases variables) and investors decision making.

## Reliability and validity of the data

We collected a sample of 552 investors, which is bigger than 300. As a result, the Shapiro-Wilk test is performed to evaluate the normality of Table 3, which shows that the data has a normal distribution. The alpha coefficients of 0.721 and 0.876 suggest a rather high level of internal consistency among the 33 items ( Table 1 and Table 2). A dependability coefficient of 0.70 or above is considered “acceptable” in most social science research situations.

**Table 1.** Cronbach’s Alpha for the market specific variables.

Cronbach’s Alpha of Reliability Coefficients for market Factors	
Cronbach’s Alpha	No. of items
0.721	24

**Table 2.** Cronbach’s Alpha for the behavioural biases’ specific variables.

Cronbach’s Alpha of Reliability Coefficients for Psychological Factors	
Cronbach’s Alpha	No. of items
0.876	20

**Table 3.** Shapiro-Wilk Test.

Decision making factors	Kolmogorov-Smirnov ^a^			Shapiro-Wilk		
	Statistic	df	Sig.	Statistic	df	Sig.
Social factors	0.244	350	0.209	0.88	350	0.261
Psychological factors	0.315	350	0.263	0.845	350	0.324

Since, both tests indicate p-values greater than 0.05 for both social and psychological components, we infer that the data does not depart significantly from normalcy, implying that normality assumptions may hold.

A descriptive and inferential analysis was conducted on all valid responses to the survey. In order to conduct a descriptive analysis, SPSS 20.0 was used. In accordance with Anderson and Garbing’s (1988) recommendation, a structural equation modelling (SEM) approach was used, and a confirmatory factor analysis was performed to assess validity of latent components, followed by a SEM analysis for hypothesis testing. This study mainly utilized the structural equation modelling (SEM) approach for two reasons: firstly, it offers a systematic way to evaluate construct-indicator linkages and model relationships within a single model ( Hair Jr *et al.*, 2017; Narasimha & Mushinada, 2020) and secondly, it provides robust and in-depth statistical approaches for dealing with financial models.

## KMO and Bartlett’s Test

The KMO value presented in Table 4. Indicates that the sample is suitable for factor analysis. A value above 0.8 is considered meritorious. Furthermore, based on the aforementioned information, the fact that the significance value is below 0.05 indicates a substantial correlation between the chosen elements in the study. The Bartlett’s Test of Sphericity has a p-value of 0.000, indicating its high statistical significance. This meets the requirement to reject the null hypothesis. It indicates that there is a good correlation between the variables, which allows for further study.

**Table 4.** KMO and Bartlett’s Test.

**Kaiser-Meyer-Olkin Measure of Sampling Adequacy**		0.864
**Bartlett’s Test of Sphericity**	**Approx. Chi-Square **	5165.365
	**df**	528
	**Sig.**	0.000

## Confirmatory factor analysis for social factors (market inefficiency)

In this study, the confirmatory analysis (CFA) is used to quantitatively evaluate validity and reliability ( Henseler, Ringle, & Sinkovics, 2009). It is often assumed and acknowledged that an item’s individual dependability is adequate if its factor loading for its primary dimensions surpasses 0.60 ( Chin *et al.*, 1997). Hair, Black, Babb, and Anderson (2010) quantified composite reliability (CR) by summing the squared factor loading and error variance of each construct. This enabled them to demonstrate that the constructions are sufficiently dependable. CR, like Cronbach’s alpha, shares the advantage of assuming equal weighting for each item in the composite load determinants, without considering the actual component loadings (Lin & Lee, 2004). Convergent validity testing involves assessing the relationship between constructs to discover if they are indeed connected. This evaluation focuses on constructs that are thought to have a connection. In contrast, discriminant validity facilitates the demonstration of the validity of constructs by establishing the absence of any association between the constructs under investigation.

It basically verifies that constructs that are not meant to be connected are not related. The overall reliability is CR > 0.70, CR > AVE, and AVE > 0.50. In the case of discriminant validity, these values should be present. MSV AVE, and the square of AVE, that is, the diagonal value, must be larger than the vertical value.

According to the established criteria, a coefficient of reliability (CR) estimates of 0.70 or greater indicates excellent reliability and demonstrates robust internal consistency ( Hair *et al.*, 2010). The composite reliability analysis ( Table 5) indicates that the assessment of all the constructs lies within the range of 0.764 to 0.862. The range exceeds the minimum requirement of 0.70, suggesting that all the metrics consistently reflect the same underlying mechanisms. The Table 5 further demonstrates the convergence validity of latent components. This study investigates the AVE (average variance extraction) values that are equal to or more than 0.50 in order to meet this criterion.

**Table 5.** Convergent validity and factor correlation matrix.

Factors	CR	AVE	MSV	MAX R (H)	Information search	Price anchoring	Herding behaviour	Confirmation bias during crisis
Information Search	0.766	0.522	0.424	0.768	0.722			
Price Anchoring	0.864	0.559	0.356	0.865	0.559	0.748		
Herding Behaviour	0.862	0.609	0.425	0.865	0.633	0.581	0.781	
Confirmation Bias during crisis	0.764	0.521	0.425	0.773	0.651	0.597	0.652	0.722

Source: author’s calculation.

The AVE was calculated by utilizing the sum of squared multiple correlations and the count of item (variables) in each comparison. All AVE values exceed 0.5, suggesting that the constructs explain more variance than measurement error and values are above 0.7, confirming good internal consistency. All constructs had satisfactory convergent validity, as shown by their convergent validity ratings of 0.50 or above ( Table 5). Hence, the constructs demonstrate good reliability and convergent validity.

**Figure 1.**
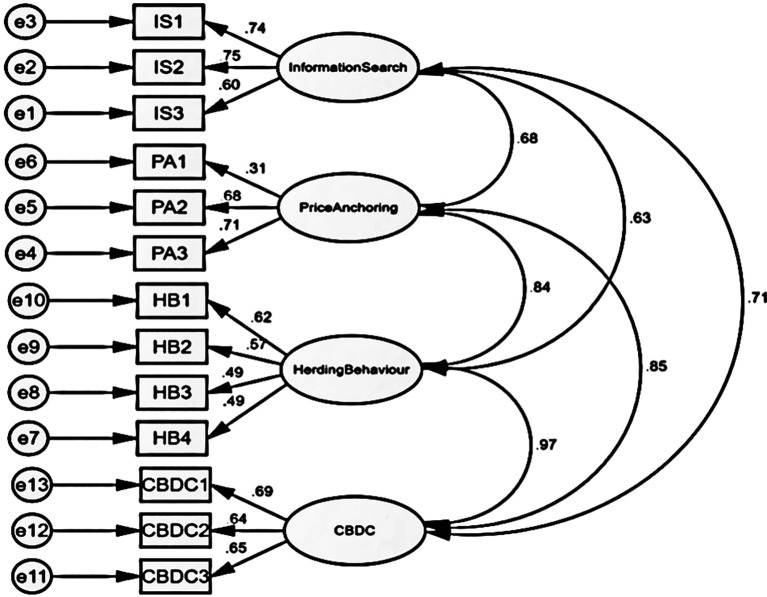
Structural model construct for market inefficiency (Social Factors).

The study employs a structural model to assess the hypothesized correlation between behavioural traits and the decision-making of individual investors. The hypothesized structural equation model was analysed to examine the goodness of fit indices and other factors related to the presented hypotheses ( Figure 1). The model fit indices presented in Table 5 indicate that the predicted structural model provides a reasonable match to the data. During the initial round of assessing fitness, the p value was found to be 0.00, indicating an exceptional degree of fitness. However, more measures were required to determine the precise fitness levels. Figure 1 illustrates the association between dependent and independent variables as indicated by the structural model.

The Table 6 above presents the model fit value for the CFA. The CMIN value of 4.022 is suitable. Yes, a GFI value of 0.947 is considered appropriate for the model. The CFI has a value of 0.966, which is considered acceptable as it above the value of GFI. The NFI result of 0.932 aligns with the model. The RMSEA value is 0.060, indicating an appropriate level. Hence, the model is a good fit.

**Table 6.** Structural model fit measure indices.

Indicator	Required for good fit	Required for acceptable fit	Obtained value
CMIN (Chi-Square/df )	0 ≤ Chi-Square/df ≤ 2	2 ≤ Chi-Square/df ≤ 5	4.022 (acceptable)
P value	0.05 ≤ p ≤ 1.00	0.01≤ p ≤ 0.05	0.046 (acceptable)
CFI	0.97 ≤ CFI ≤ 1.00	0.95 ≤ CFI ≤ 0.97	0.966 (acceptable)
GFI	0.95 ≤ GFI ≤ 1.00	0.90 ≤ GFI ≤ 0.95	0.947 (acceptable)
AGFI	0.90 ≤ AGFI ≤ 1.00	0.85 ≤ GFI ≤ 0.90	0.924 (good)
NFI	0.95 ≤ NFI ≤ 1.00	0.90 ≤ NFI ≤ 0.95	0.932 (acceptable)
RMSEA	< 0.05	<.08	0.060 (acceptable)
TLI	>.9	<5.0	0.921 (good)

Source: author’s calculation.

**Figure 2.**
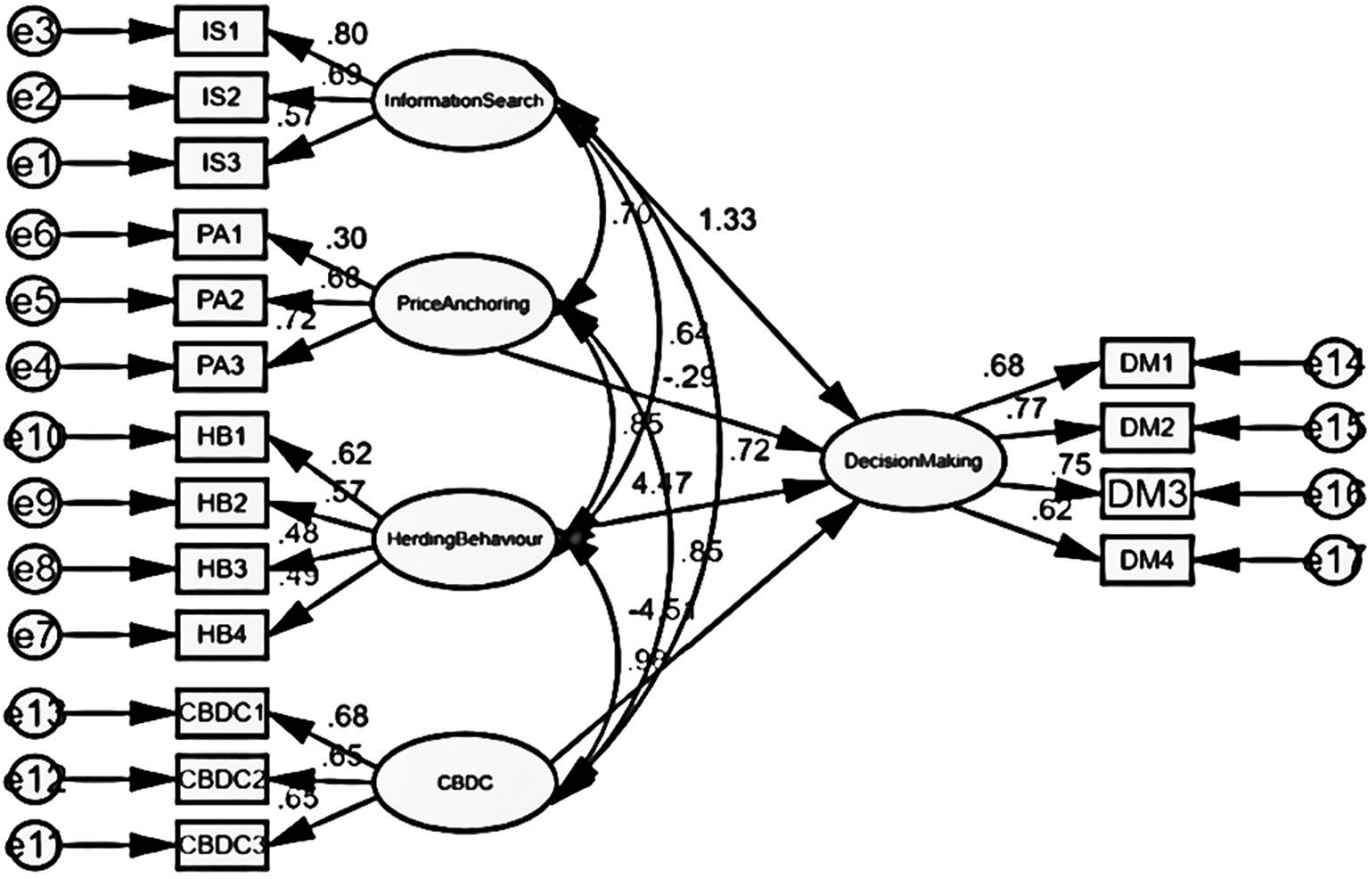
Decision making structural model (social factors).

This study employed a structural model to assess the hypothesized correlation between socioeconomic variables and the investment choices made by individual investors. Various indications of goodness of fit are observed for the specified hypotheses in the structural model depicted in Figure 2, with a focus on exploring the parameters. Table 7 displays the findings of the model fit indices, indicating that the suggested structural model effectively explains a strong correlation between statistical and social variables. Therefore, the null hypothesis has been rejected. The model has an acceptable to good fit, with certain indices meeting high requirements and others falling under acceptable ranges.

**Table 7.** Structural model fit measure indices for decision making.

Indicator	Required for good fit	Required for acceptable fit	Obtained value
CMIN (Chi-Square/df )	0 ≤ Chi-Square/df ≤ 2	2 ≤ Chi-Square/df ≤ 5	3.569 (acceptable)
P value	0.05 ≤ p ≤ 1.00	0.01≤ p ≤ 0.05	0.071 (good)
CFI	0.97 ≤ CFI ≤ 1.00	0.95 ≤ CFI ≤ 0.97	0.956 (acceptable)
GFI	0.95 ≤ GFI ≤ 1.00	0.90 ≤ GFI ≤ 0.95	0.933 (acceptable)
AGFI	0.90 ≤ AGFI ≤ 1.00	0.85 ≤ GFI ≤ 0.90	0.920 (good)
NFI	0.95 ≤ NFI ≤ 1.00	0.90 ≤ NFI ≤ 0.95	0.912 (acceptable)
RMSEA	< 0.05	<.08	0.011 (good)
TLI	>.9	<5.0	0.931 (good)

Source: author’s calculation.

Table 8 illustrates the correlation between factors contributing to market inefficiency and the process of decision making. The study found that Information Search, Price Anchoring, Herding Behaviour, and CBDC had significant impacts, with β coefficients of 1.73 (p = 0.017), -0.318 (p = 0.023), 6.345 (p = 0.030), and -0.464 (p = 0.012) respectively. In each example, the value of P is below 0.05, showing a statistically significant link. Hence, social influences exert influence on investors’ decision-making process. The model demonstrates an **acceptable to good fit**, with some indices achieving high standards while others remain within acceptable limits.

**Table 8.** Relationship between market inefficiency and decision making.

Relationship			Estimate	S.E.	C.R.	P
Decision Making	<---	Information Search	1.738	0.728	2.386	0.017
Decision Making	<---	Price Anchoring	-0.318	1.048	-0.304	0.023
Decision Making	<---	Herding Behaviour	6.345	3.497	1.815	0.030
Decision Making	<---	CBDC	-4.646	2.760	-1.683	0.012

Source: author’s calculation.

**Figure 3.**
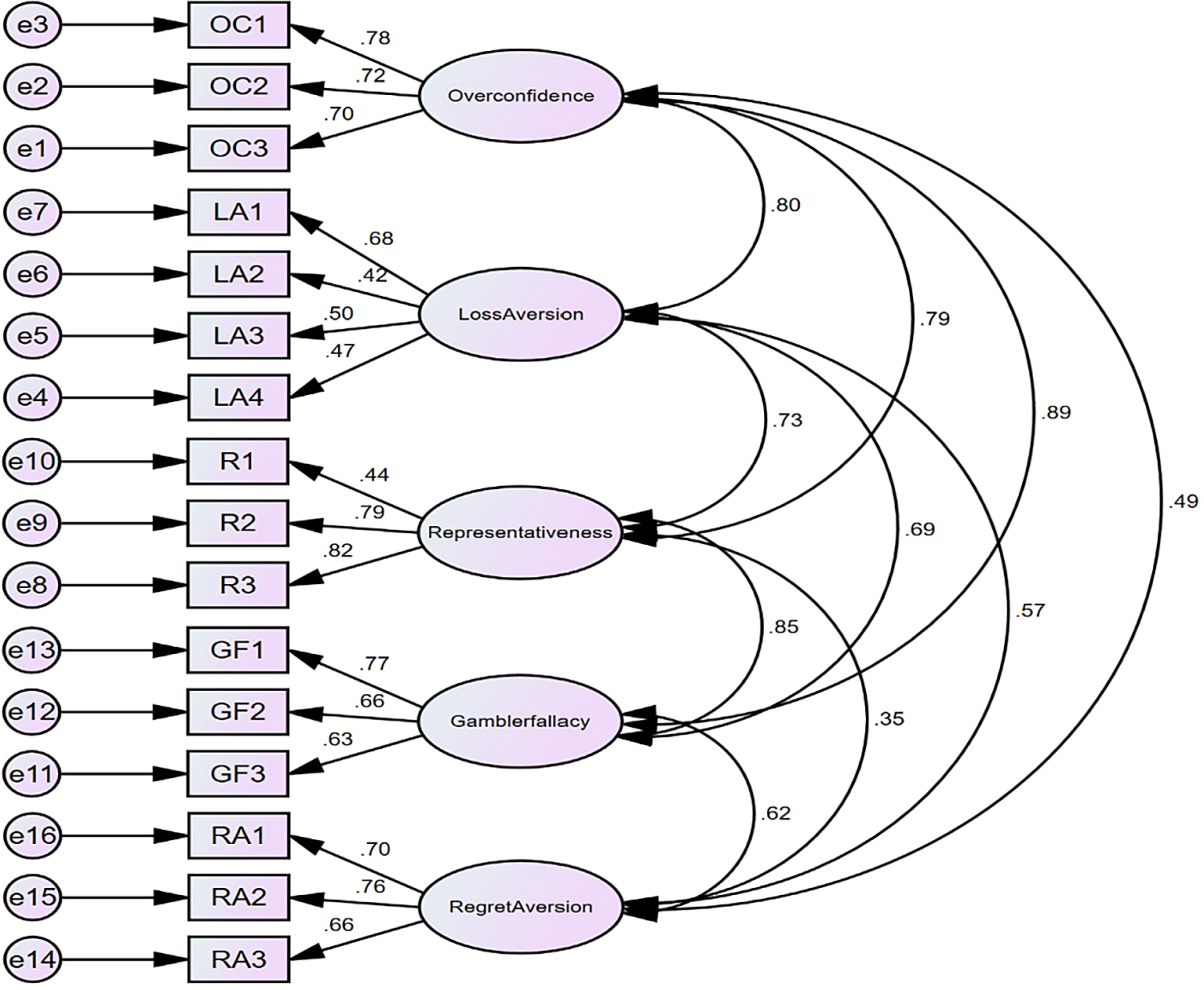
Model for market behavioural Biases (Psychological factors).

**Interpretation through Behavioural Finance Theories**

a) **Information Search (Estimate: 1.738, p = 0.017)**
Significant positive impact suggests that greater information search enhances decision-making quality. Consistent with ***Prospect Theory***, individuals gather more information to reduce perceived risks and mitigate loss aversion effects.
b) **Price Anchoring (Estimate: -0.318, p = 0.023)**
The negative coefficient suggests that reliance on initial price anchors hinders rational decision-making. Supports ***Anchoring** Bias* from ***Cognitive Bias Theory***, where individuals irrationally fixate on reference points, leading to suboptimal choices.
c) **Herding Behaviour (Estimate: 6.345, p = 0.030)**
Strong positive influence, indicating that collective behaviour significantly shapes financial decisions. Aligns with ***Herding Theory*** in behavioural finance, where social influence and fear of missing out (FOMO) drive decision-making.
d) **Confirmation Bias during Crisis (Estimate: -4.646, p = 0.012)**
Negative effect implies that during crises, confirmation bias leads to poor financial choices. Related to *Cognitive Dissonance Theory*, where individuals selectively seek information that aligns with pre-existing beliefs, reinforcing suboptimal financial behaviour.

## Confirmatory factor analysis for psychological factors

The findings regarding composite reliability, as presented in Table 9, indicate that the evaluation of all constructs varies between 0.712 and 0.778. This significantly surpasses the minimum threshold of 0.70 and signifies that all measures consistently capture the same latent constituencies as it is reflected in Figure 3. The Table 9 demonstrates the convergent validity of latent variables. All constructs have CR > 0.7, indicating good reliability. AVE scores equal to or greater than 0.50. Sarstedt *et al.* (2014) examined for this study in order to meet this criterion. Investors often lack widespread logic while making investment judgments. The outcomes of the current investigation corroborated this concept. Investors’ financial behaviour is determined by their cognitive capacity to understand all pertinent facts. Their choice of investing opportunities and assets is impacted by their preexisting views, previous experiences, and market trends. Rather of scrutinizing existing information, investors solicit guidance from acquaintances, coworkers, and brokers. Investors admit that their financial decisions are impacted by emotions and ambitions. Gaining a comprehensive understanding of various behavioural biases is crucial for managing and mitigating these biases, hence improving investment performance.

**Table 9.** Convergent validity and factor correlation matrix.

Factors	CR	AVE	MSV	MAX R (H)	Over-confidence	Loss aversion	Representativeness	Gambler’s fallacy	Regret aversion
Overconfidence	0.796	0.422	0.324	0.668	0.712				
Loss aversion	0.894	0.459	0.456	0.765	0.559	0.728			
Representativeness	0.872	0.509	0.325	0.765	0.633	0.581	0.778		
Gambler’s fallacy	0.864	0.521	0.425	0.773	0.651	0.597	0.652	0.732	
Regret aversion	0.864	0.521	0.425	0.673	0.651	0.597	0.652	0.713	0.722

Source: author’s calculation.

Table 10 displays the model fit value for the CFA. The CMIN score of 2.021 is acceptable. Indeed, the GFI value of 0.937 is appropriate for the model. CFI has a value of 0.974, which is acceptable because it is larger than GFI. The NFI value of 0.918 agrees with the model. The RMSEA value is 0.0964, which is good. Thus, the model fits flawlessly.

**Table 10.** Structural model fit measure indices (Psychological factors).

Indicator	Required for good fit	Required for acceptable fit	Obtained
CMIN (Chi-Square/df )	0 ≤ Chi-Square/df ≤ 2	2 ≤ Chi-Square/df ≤ 5	2.021 (Acceptable fit)
P value	0.05 ≤ p ≤ 1.00	0.01≤ p ≤ 0.05	0.061 (Good Fit)
CFI	0.97 ≤ CFI ≤ 1.00	0.95 ≤ CFI ≤ 0.97	0.974 (Good Fit)
GFI	0.95 ≤ GFI ≤ 1.00	0.90 ≤ GFI ≤ 0.95	0.937 (Acceptable fit)
AGFI	0.90 ≤ AGFI ≤ 1.00	0.85 ≤ GFI ≤ 0.90	0.912 (Good Fit)
NFI	0.95 ≤ NFI ≤ 1.00	0.90 ≤ NFI ≤ 0.95	0.918 (Acceptable fit)
RMSEA	< 0.05	<.08	0.069 (Acceptable fit)
TLI	>0.9	<5.0	0.964 (Good Fit)

Source: author’s calculation.

In addition to these important findings, this study shows that Indian investors are not completely rational and that the country’s marketplaces are inefficient. The association between these findings and the investors’ decision-making process was evaluated empirically by interviewing the investors.

## Findings

**Figure 4.**
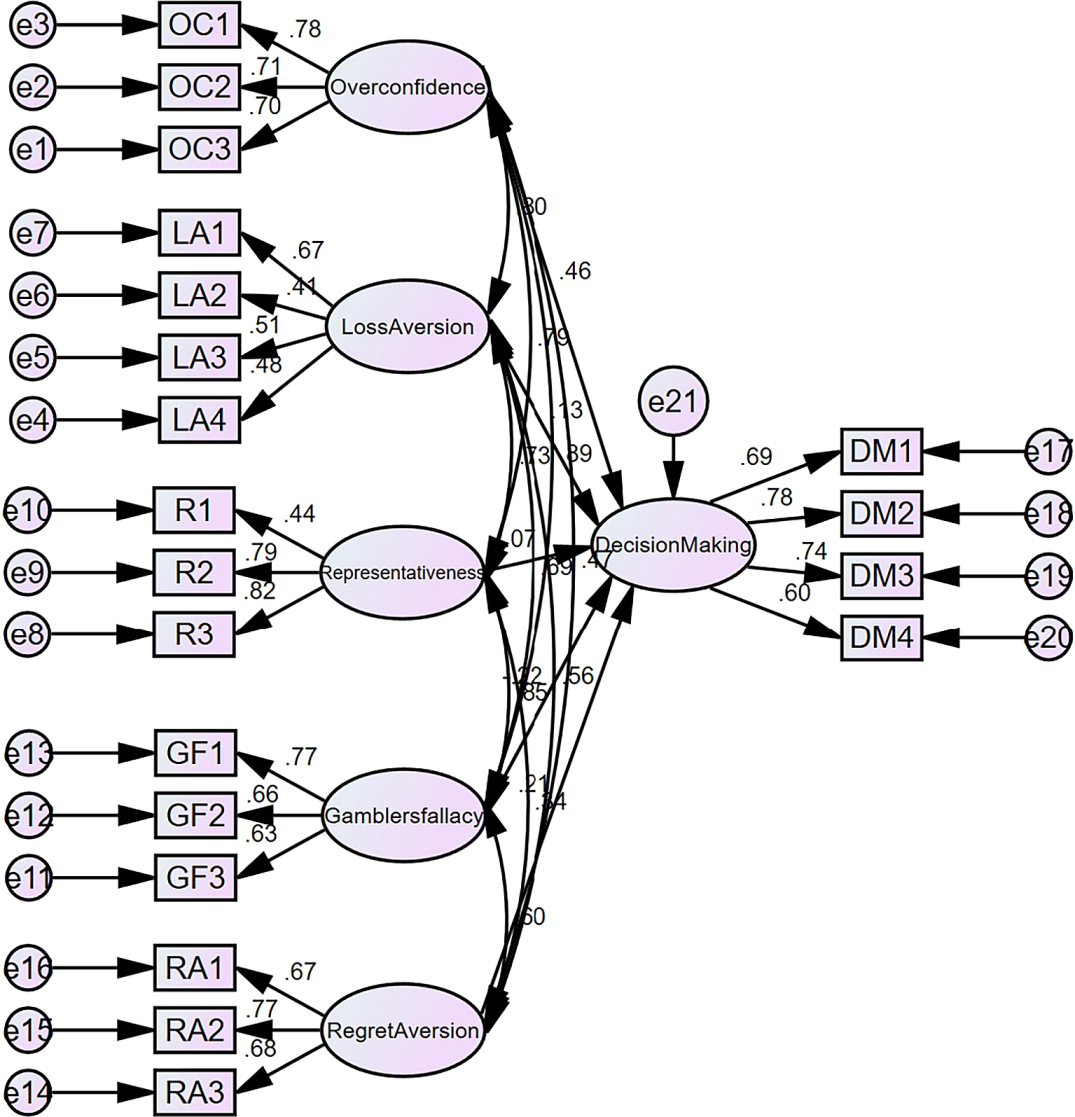
Decision making structural model (behavioural biases).

Tables 11 and 12 illustrates the correlation amongst behavioural biases and financial decision making. The effects of overconfidence, loss aversion, representativeness, gambler’s fallacy, and regret aversion the values of β and p for the given data are as follows: β = 0.534 with p = 0.036, β = 0.202 with p = 0.003, β = 0.071 with p = 0.047, and β = -0.249 with p = 0.024. The P value in each example is below 0.05, showing a statistically significant link. Hence, psychological elements exert influence on investors’ decision-making process ( Figure 4). With respect to the dependent and independent variables, Table 12 presents the outcomes of the structural model. Cognitive biases include overconfidence, loss aversion, representativeness, the gambler’s fallacy, and regret aversion affect investors’ decisions. The results of the research indicate that individual stock market participants in India employ implicit biases to accelerate their decision-making when influenced by behavioural biases. This study’s results suggest that behavioural biases have a substantial influence on the decision-making process of Indian investors. Regarding social impact, herding has been identified as a very important factor, indicating that investors tend to imitate the investment behaviours of their peer groups.

**Table 11.** Structural model fit measure indices.

Indicator	Required for good fit	Required for acceptable fit	Obtained
CMIN (Chi-Square/df )	0 ≤ Chi-Square/df ≤ 2	2 ≤ Chi-Square/df ≤ 5	2.569 (Acceptable fit)
P value	0.05 ≤ p ≤ 1.00	0.01≤ p ≤ 0.05	0.069 (Good Fit)
CFI	0.97 ≤ CFI ≤ 1.00	0.95 ≤ CFI ≤ 0.97	0.966 (Acceptable fit)
GFI	0.95 ≤ GFI ≤ 1.00	0.90 ≤ GFI ≤ 0.95	0.965 (Good Fit)
AGFI	0.90 ≤ AGFI ≤ 1.00	0.85 ≤ GFI ≤ 0.90	0.946 (Good Fit)
NFI	0.95 ≤ NFI ≤ 1.00	0.90 ≤ NFI ≤ 0.95	0.967 (Good Fit)
RMSEA	< 0.05	<.08	0.071 (Acceptable fit)
TLI	>.9	<5.0	0.814 (Acceptable fit)

(Psychological factors and decision making).

Source: author’s calculation.

**Table 12.** Relationship between psychological factors and decision making.

Relationship between Psychological Factors (Behavioural Biases) and Decision Making			Estimate	S.E.	C.R.	P
Decision Making	<---	Overconfidence	0.534	0.686	0.779	0.036
Decision Making	<---	Loss Aversion	0.202	0.671	0.301	0.003
Decision Making	<---	Representativeness	0.071	0.456	0.155	0.047
Decision Making	<---	Gambler’s fallacy	-0.249	0.956	-0.261	0.024
Decision Making	<---	Regret Aversion	0.210	0.317	0.663	0.007

(Behavioural Biases) and Decision-Making.

Source: author’s calculation.

**Interpretation with Behavioural Finance Theories**

a) **Overconfidence (β = 0.534, p = 0.036)** aligns with the **Prospect Theory** and **Self-attribution Bias**, suggesting that investors overestimate their knowledge, leading to excessive trading or risk-taking.
b) **Loss Aversion (β = 0.202, p = 0.003)** strongly supports **Prospect Theory**, where investors prefer avoiding losses over making equivalent gains.
c) **Representativeness (β = 0.071, p = 0.047)** connects to **Heuristic-driven Bias**, where individuals rely on past patterns rather than rational decision-making.
d) **Gambler’s Fallacy (β = -0.249, p = 0.024)** is associated with **Hot-Hand Fallacy**, indicating irrational predictions based on past outcomes, affecting trading behaviour.
e) **Regret Aversion (β = 0.210, p = 0.007)** ties to **Regret Theory**, where investors avoid actions that could lead to future regret, influencing risk tolerance.

This study confirms the results of Chen, Rui, and Xu (2003), who demonstrated that the phenomenon of herd behaviour is more widespread in developing markets compared to developed market. An investor’s decision-making process is impacted by many psychological biases, such as overconfidence, loss aversion, representativeness, the gambler’s fallacy, and regret aversion. According to the results, Indian investors are highly influenced by their own behavioural biases when making investment decisions. One of the most powerful forms of social influence is herding, which means that people tend to mimic the investment strategies used by their friends and colleagues.

## Conclusion

This study confirms that behavioural biases play a significant role in shaping investment decisions, particularly in emerging markets such as India. The findings align with previous research, notably Chen, Rui, and Xu (2003); Li and Xing (2023); Das and Panja (2024) highlighting the prevalence of herd behaviour in developing economies. Indian investors exhibit strong cognitive biases, including overconfidence, loss aversion, representativeness, the gambler’s fallacy, and regret aversion, all of which significantly impact their financial decision-making process. The statistical results further reinforce the substantial influence of these biases, underscoring the role of psychological factors in investment behaviour. Although risk and return are commonly used to evaluate investment performance, this metric does not consistently demonstrate its ability to anticipate financial returns and investment choices for investors. While classical theories may not always accurately predict market conditions, it is appropriate to acknowledge the significance of behavioural biases in investment decision-making. Our findings are consistent with the previous research based upon the investors decision making factors ( Raut *et al.*, 2018). The main aim of this study is to determine the importance of socio-psychological factors in affecting the decision-making behaviour of individual investors.

In order to make rational investing decisions, this study shows which socio-psychological biases are most common. Based on the results of this study, it is critical for fund managers to be able to identify and avoid making the same mistakes again when it comes to monitoring. Therefore, fund managers should utilize a flexible investing approach in an intricate market system where individual emotions have a substantial impact on developing an effective portfolio. This study provides useful insight into the most prevalent socio-psychological factor that should be considered by investors when determining a suitable portfolio level. From a management perspective, it is imperative that fund managers possess the ability to identify and avoid reiterating common behavioural mistakes, as indicated by the study’s results. These implications can provide guidance to investment advisors, regulators, and other players in the market, as well as to individual investors, prompting them to reflect on their investment-making choices throughout different time periods.

The implications of these findings extend beyond individual investors to fund managers, investment advisors, and policymakers. Recognizing and mitigating the impact of cognitive biases can lead to more rational investment decisions and better portfolio management. Fund managers, in particular, should adopt a flexible investment approach that accounts for emotional influences, reducing the likelihood of recurring behavioural mistakes. Furthermore, market regulators and policymakers can use these insights to develop investor education programs and regulatory frameworks that minimize the adverse effects of herd behaviour and cognitive biases.

Ultimately, while traditional financial models emphasize risk and return, this study highlights the need to incorporate behavioural finance principles into investment strategies. By acknowledging and addressing the psychological drivers of investment behaviour, investors and financial professionals can enhance decision-making processes, leading to more stable and efficient financial markets.

Future research can examine scenarios or surroundings in which investor-specific factors, such as the behavioural finance approach, dominate one another. Researchers can also analyse the predominant market-related drivers that have the biggest influence on investor sentiments across time, in different scenarios, and within various country contexts. The limited influence of macroeconomic fundamentals and policy changes may also be a compelling subject for future scholars to investigate.

### Recommendations

This study sheds light on how behavioural biases and social influence affect investment decision-making, notably in the Indian stock market. Given the importance of cognitive biases such as overconfidence, loss aversion, representativeness, gambler’s fallacy, and regret aversion, investors, fund managers, and policymakers must consider these psychological variables when developing investment tactics and regulatory frameworks.

Furthermore, the study underlines the impact of herding behaviour among Indian investors, emphasizing the significance of investor education activities. Regulatory agencies can take proactive steps to reduce excessive herding by supporting financial literacy programs that encourage independent and well-researched investing decisions. Implementing market laws targeted at increasing openness and lowering information asymmetry can help to lessen the hazards associated with herd behaviour.

### Ethics and consent

This research adhered to the criteria set by the Uttaranchal University Research Ethics Board (REB), which granted ethics approval under number UU/DRI/EC/2024/007 (18th Sept. 2024). The formal approval letter was issued retroactively. In July 2024, we obtained verbal permission from the REB to commence our study, as written approval is provided only when requested by a journal. The REB reviewed our comprehensive application, confirming that we met all ethical norms and regulations before granting preliminary verbal consent. Consequently, we initiated our investigation in good faith, fully aligned with the ethical standards outlined in the preliminary review.

The questionnaire was submitted to the REB of the university, where board members and the chairperson assessed the viability of the research topic. After all members presented their research objectives, the questionnaire was approved for conducting the study.

### Consent statement

All of the study’s participants have provided written informed consent. A self-explanatory written statement was included with the questionnaire for participants, and a similar questionnaire was submitted to the University research board.

## Data Availability

Figshare: Decoding investor sentiments in the Indian stock market: a structural equation modelling approach, https://doi.org/10.6084/m9.figshare.27021937 ( Farman Ali *et al.*, 2024). Data are available under the terms of the Creative Commons Attribution 4.0 International license (CC-BY 4.0).
