# Supplementary material for: Decoding Investor Sentiments in the Indian Stock Market: A Structural Equation Modelling Approach
Source: F1000Res. 2025 May 2;13:1378. Originally published 2024 Nov 18. [Version 2] doi: 10.12688/f1000research.156635.2 (PMC12107233; doi:10.12688/f1000research.156635.2)
Supplement: Peer review history

# Reviewer report: Reviewer response for version 2

**DOI:** 10.5256/f1000research.179493.r345422

**Published:** 2025-10-06

**Authors:** Poshan Yu

**Affiliations:** 1 Soochow University, Suzhou, Jiangsu, China

After evaluating “Decoding Investor Sentiments in the Indian Stock Market: A Structural Equation Modelling Approach,” I recommend acceptance for indexing. The study delivers meaningful value to behavioral finance, with key strengths justifying this decision:​

 1. Critical Focus on an Understudied Context​

 By centering the Indian stock market—an emerging economy with growing retail investor participation—the study fills a gap in Western-dominated behavioral finance research. Its focus on how psychological biases shape local investors is academically relevant and practically useful for regulators/advisors in India.​

 2. Rigorous Methodology​

 A nationally representative dataset of 552 retail investors, paired with Structural Equation Modelling (SEM) (ideal for testing complex bias-sentiment relationships), ensures the study’s findings are credible and generalizable.​

 3. Impactful Findings​

 The study’s conclusions—linking biases (anchoring, herding, overconfidence) to investor irrationality and market inefficiency—advance knowledge by validating behavioral theories in a non-Western context. It also paves the way for cross-country comparisons or policy-focused follow-up research.​

 Minor Refinement (Optional)​

 Briefly contrasting findings with other emerging markets could enhance depth, but this does not affect acceptance.​

 Conclusion​

 This study is a strong addition to behavioral finance. Its relevance, rigor, and novel insights make it worthy of indexing.

Is the work clearly and accurately presented and does it cite the current literature?

Yes

If applicable, is the statistical analysis and its interpretation appropriate?

Yes

Are all the source data underlying the results available to ensure full reproducibility?

Yes

Is the study design appropriate and is the work technically sound?

Yes

Are the conclusions drawn adequately supported by the results?

Yes

Are sufficient details of methods and analysis provided to allow replication by others?

Yes

Reviewer Expertise:

M&A, Finance, ESG and FinTech

I confirm that I have read this submission and believe that I have an appropriate level of expertise to confirm that it is of an acceptable scientific standard.

# Reviewer report: Reviewer response for version 2

**DOI:** 10.5256/f1000research.179493.r383241

**Published:** 2025-05-27

**Authors:** Dr. Mohammad Nayeem Abdullah

**Affiliations:** 1 Chittagong Independent University, Chittagong, Chittagong Division, Bangladesh

**Introduction**: Research gap left out by the previous authors should be more specifically   mentioned. A statement explaining that the research findings are different from previous studies should be provided.  A brief statement about the significance or contribution of the study should be included. State how the paper is organized into different sections and what they are about.

**Literature review & hypothesis:**

 Research question or statement of problems and hypotheses should be drawn on the basis of the weakness/drawbacks of the previous studies. That is, a statement describing how previous studies did not address the current issue of research should be provided.

**Methodology: **Explain how the methods are suitable to answer the research question or solve the stated problem and test the hypotheses.

**Discussion: **The paper explains:

1. how the results are related to RQ and objectives: Moderately
2. whether the results supported or contradicted previous theories or findings? Weak (Need to be improved)
3. How the how the research has provided new knowledge. (Need to be more precise)
4. the significance and implication of your results. (It’s ok)

 limitations and scope for further research should be mentioned.

**Empirical findings are fine. With those minor revisions paper can be indexed.**

Is the work clearly and accurately presented and does it cite the current literature?

Partly

If applicable, is the statistical analysis and its interpretation appropriate?

Partly

Are all the source data underlying the results available to ensure full reproducibility?

Yes

Is the study design appropriate and is the work technically sound?

Yes

Are the conclusions drawn adequately supported by the results?

Partly

Are sufficient details of methods and analysis provided to allow replication by others?

Partly

Reviewer Expertise:

Corporate Social Responsibility, Banking & capital market issues.

I confirm that I have read this submission and believe that I have an appropriate level of expertise to confirm that it is of an acceptable scientific standard.

# Reviewer report: Reviewer response for version 2

**DOI:** 10.5256/f1000research.179493.r382122

**Published:** 2025-05-26

**Authors:** Dipendra Karki

**Affiliations:** 1 Tribhuvan University, Kirtipur, Central Development Region, Nepal

Upon reviewing the revised manuscript, I found that some issues have been adequately addressed, while others are only partially addressed. For example, the paper now properly includes the organization of the study. Limitations of the study also have been incorporated.

 However, it developed and tested only one hypothesis, with no distinct hypotheses for social and psychological biases.

 Most of the missing references for citations are included and formatted in APA style, although a few are still absent, such as (C.V., 2017).

 The sampling process and sample strata for the heterogeneous group are now discussed. Empirical evidence establishes the validity of the results and addresses cross-validation issues.

 The determination of alpha values for social and psychological factors is unclear (Tables 1 and 2, both with d.f. 350?, Table 3). Aligning market factors with social factors, which have alpha values of 0.721 (Table 1), is necessary.

 Results are enhanced by separately estimating the influence of social and psychological elements on investment decisions using confirmatory factor analysis (CFA). It seems to be fine.

 The conclusion has been restructured and is based on the findings.

 To sum up, the manuscript presents relevant issues within behavioral finance and reflects notable improvements. However, some theoretical and methodological reservations remain as stated. Based on these issues, I approve the manuscript with reservations.

**Dipendra Karki, PhD**

 Reviewer

Is the work clearly and accurately presented and does it cite the current literature?

Partly

If applicable, is the statistical analysis and its interpretation appropriate?

No

Are all the source data underlying the results available to ensure full reproducibility?

Partly

Is the study design appropriate and is the work technically sound?

No

Are the conclusions drawn adequately supported by the results?

No

Are sufficient details of methods and analysis provided to allow replication by others?

Partly

Reviewer Expertise:

My area of expertise is finance, with a special emphasis on stock market research, investing methods, and financial economics, particularly behavioral finance.

I confirm that I have read this submission and believe that I have an appropriate level of expertise to confirm that it is of an acceptable scientific standard, however I have significant reservations, as outlined above.

# Reviewer report: Reviewer response for version 2

**DOI:** 10.5256/f1000research.179493.r382121

**Published:** 2025-05-19

**Authors:** Umar Kayani

**Affiliations:** 1 Al Ain University, Al Ain, Abu Dhabi, United Arab Emirates

No further comments. Thank you.

Is the work clearly and accurately presented and does it cite the current literature?

Partly

If applicable, is the statistical analysis and its interpretation appropriate?

Yes

Are all the source data underlying the results available to ensure full reproducibility?

No

Is the study design appropriate and is the work technically sound?

Partly

Are the conclusions drawn adequately supported by the results?

Partly

Are sufficient details of methods and analysis provided to allow replication by others?

Partly

Reviewer Expertise:

Fintech, crypto currencies, bitocin, coroporate finance, corporate governance, environmental issues

I confirm that I have read this submission and believe that I have an appropriate level of expertise to confirm that it is of an acceptable scientific standard.

# Reviewer report: Reviewer response for version 1

**DOI:** 10.5256/f1000research.171967.r359169

**Published:** 2025-02-13

**Authors:** Rodiat Lawal

**Affiliations:** 1 University of Dundee, Dundee, Scotland, UK

Summary of the paper:

 This study investigates the impact of psychological and social biases on retail investors in the Indian stock market using Structural Equation Modelling (SEM). The research is based on a survey of 552 investors and examines how cognitive biases such as overconfidence, herding behaviour, anchoring, and loss aversion influence investment decisions, potentially leading to market inefficiencies. The study contributes to the field of behavioural finance by highlighting how investor sentiment can distort financial decision-making and asset pricing in an emerging market.

 The findings suggest that psychological biases significantly influence investment behaviour, reinforcing the idea that markets are not fully efficient. The study further implies that policy interventions and investor education programs could help mitigate the negative effects of these biases.

 Questions evaluation

 1. Clarity and Accuracy of Presentation (Partly)

 The research question is clearly defined, and the introduction provides sufficient background.

 The structure follows a logical progression: introduction → literature review → methodology → results → discussion → conclusion. The figures and tables are well-presented and support the findings.

 However, overly complex sentences and wordiness reduce readability. Some sentences should be restructured for clarity and conciseness. Also, redundancy in the literature review should be addressed by consolidating similar studies and focusing more on their relevance. Some sections lack clear transitions, making it harder for readers to follow the argument.

 Specific Recommendation:

 a. Revise for conciseness and clarity by avoiding long, complex sentences.

 Example of a revision:

*Current:* “Investor sentiment significantly influences financial markets, frequently causing stock prices to deviate from their intrinsic values.”

*Suggested:* “Investor sentiment affects financial markets by driving stock prices away from intrinsic values.”

 2. Citation of Current Literature (Partly)

 The literature review includes a wide range of sources, covering both classical finance theories (Efficient Market Hypothesis, Random Walk Theory, etc.) and behavioural finance theories.

 The study cites seminal works in behavioural finance, including De Long et al. (1990), Kumar & Mahakud (2016), and Wen et al. (2019).

 The study relies heavily on older literature and does not sufficiently engage with recent studies (2020–2024). Some sources are cited multiple times unnecessarily, adding redundancy. More India-specific studies should be included to strengthen the contextual relevance.

 3. Methodological Details and Replicability

 The study uses Structural Equation Modelling (SEM), which is an appropriate technique for evaluating multiple relationships among latent variables. A sample of 552 investors is robust and provides a solid foundation for statistical inference.

 However, the justification for using SEM over other statistical methods (e.g., regression analysis, factor analysis) is weak. There is limited discussion on potential biases in the sample selection and how they were controlled. The survey design and question structure are not detailed enough to allow full replication. The limitations of SEM (e.g., sensitivity to multicollinearity, model fit issues) are not discussed.

 The authors should: 

 a. Clearly justify why SEM was chosen over alternative methods.

 b. Provide more details on the survey structure (e.g., how questions were designed, sample recruitment, response rates).

 c. Discuss potential biases (e.g., self-selection bias, response bias) and their implications for results.

 d. Include a brief limitations section highlighting the constraints of the SEM model.

 4. Statistical Analysis and Interpretation (Partly)

 The study presents standard SEM model fit indices (CFI, GFI, RMSEA, etc.).  As such the statistical analysis is largely appropriate for the research questions.

 However, Some model fit indices (e.g., RMSEA = 0.069) slightly exceed recommended thresholds. There is no discussion on multicollinearity or alternative model specifications.

 Also, the interpretation of results is too descriptive-it needs stronger theoretical links to behavioural finance concepts.

 The authors should: 

 a. Provide a justification for the acceptability of model fit indices when they slightly exceed thresholds.

 b. Include multicollinearity checks to ensure validity.

 c. Strengthen the interpretation of findings by explicitly linking results to behavioural finance theories.

 5. Conclusion and Policy Implications (Partly)

 The conclusions generally align with the results and highlight the role of investor sentiment in market inefficiency.

  However, the conclusions could be more specific—they currently restate findings without deeper analysis. Policy implications are vague and need clear, actionable recommendations.

 Authors should:

 a. Expand on practical applications e.g., how financial advisors, policymakers, or retail investors can use these insights.

 b. Provide specific recommendations (e.g., financial literacy programs, sentiment-tracking tools for investors).

Is the work clearly and accurately presented and does it cite the current literature?

Partly

If applicable, is the statistical analysis and its interpretation appropriate?

Partly

Are all the source data underlying the results available to ensure full reproducibility?

Yes

Is the study design appropriate and is the work technically sound?

Partly

Are the conclusions drawn adequately supported by the results?

Partly

Are sufficient details of methods and analysis provided to allow replication by others?

Partly

Reviewer Expertise:

Corporate Finance, International Business, Corporate Governance, Behavioural Finance, and Sustainable Finance.

I confirm that I have read this submission and believe that I have an appropriate level of expertise to confirm that it is of an acceptable scientific standard, however I have significant reservations, as outlined above.

# Reviewer report: Reviewer response for version 1

**DOI:** 10.5256/f1000research.171967.r345421

**Published:** 2025-02-13

**Authors:** Umar Kayani

**Affiliations:** 1 Al Ain University, Al Ain, Abu Dhabi, United Arab Emirates

While some background is necessary, the introduction is overly detailed and could be more concise. In the introduction section, the authors shortly explain the contribution of this study. However, towards the end of the introduction section, the remaining contributions are outlined. The authors are advised to adhere to the standard procedure of explaining relevant key factors in specified sections.

 The authors are What is the motivation behind conducting this study, and particular this estimation model. Why was the this estimation model selected, and on what basis? The author needs to provide some preliminary analysis to demonstrate the best fit of the model and the grounds on which this specific estimation model was selected.

 I suggest adding a literature review section to support critical discussion on results, study limitations, and future agendas within the scope of similar studies in the same area.

 Additionally, clearly stating the contribution of the study is essential. The literature review should explicitly identify the research gap or question that the current study aims to address, setting the stage for the study's objectives and contributions. Support your statement with citations from previous similar studies. Many abbreviations are used in this study. It would be beneficial to create a table explaining all the abbreviations used. See some of studies as below. (Refer 1 and 2)

 In the results section, there is no discussion explaining the behaviour of the variable the author needs to explain the results. The discussion mentions results from previous studies but does not contextualize these findings within the scope of the current research. How do these results relate to the specific findings in accordance with this study?

 The study does not elaborate on the practical implications of these findings. How can investors and policymakers use this information to make informed decisions?

 The suggestion for future research is missing. Providing more focused and actionable suggestions for future research would be beneficial. The conclusion could benefit from a more organized structure, with clear subsections summarizing specific aspects of the research, such as findings, implications, limitations, and future research directions.

 Finally, the article needs some language corrections. I believe these comments will help to improve the current version of the manuscript.

Is the work clearly and accurately presented and does it cite the current literature?

Partly

If applicable, is the statistical analysis and its interpretation appropriate?

Yes

Are all the source data underlying the results available to ensure full reproducibility?

No

Is the study design appropriate and is the work technically sound?

Partly

Are the conclusions drawn adequately supported by the results?

Partly

Are sufficient details of methods and analysis provided to allow replication by others?

Partly

Reviewer Expertise:

Fintech, crypto currencies, bitocin, coroporate finance, corporate governance, environmental issues

I confirm that I have read this submission and believe that I have an appropriate level of expertise to confirm that it is of an acceptable scientific standard, however I have significant reservations, as outlined above.

## References

[1] : Investor sentiment and stock price crash risk: The mediating role of analyst herding. *Computers in Human Behavior Reports* .2024;13: 10.1016/j.chbr.2024.100371 10.1016/j.chbr.2024.100371

[2] : Sustainable investing: ESG effectiveness and market value in OECD regions. *Cogent Economics & Finance* .2025;13(1) : 10.1080/23322039.2024.2445147 10.1080/23322039.2024.2445147

# Reviewer report: Reviewer response for version 1

**DOI:** 10.5256/f1000research.171967.r345427

**Published:** 2024-12-27

**Authors:** Dipendra Karki

**Affiliations:** 1 Tribhuvan University, Kirtipur, Central Development Region, Nepal

**Review Report**

 Manuscript Title: **Decoding Investor Sentiments in the Indian Stock Market: A Structural Equation Modelling Approach**

**Abstract/Introduction:**

 The study primarily aims to analyse the impact of social biases and psychological bases on investors’ decision-making. However, the proxy variables for the latent factor, 'social biases,’ are not found sufficient to capture the social context. It seems the study has used information search (IS), Price Anchoring (PA), Heuristic Behavior (HB), & CBDC as the proxy for social factors, which the theory doesn’t support. They rather fall in the behavioural domain. It is required to include the theoretical bases for considering those factors as social biases in the study. 

 Further, the study develops only one hypothesis related to psychological (behavioural) biases and decision-making, missing to address the social biases. Though it showed that both hypotheses were tested. This brings significant discrepancy with the study objectives.

 Need to identify the main research issues and problems and mention how this study addresses these problems.

 Though it addresses some highly interesting issues, highlighting the limitations of this study would be better to hint the future researchers, giving further scope of the study in this area.

 The last paragraph is expected to discuss about the organization of the study. How the subsequent sections have been arranged to make readers acquainted with the flow of the study.

**Literature Review:**

 The literature on social factors is not sufficiently reviewed to establish the validity of employed observed variable ‘information seeking’ as mostly the reviews are made on psychological factors.

 Some citations, such as (C.V, 2017) are not included in the reference/bibliography list, making it difficult to verify the sources or their relevance.

 Additionally, few in-text citations [e.g., S. Kumar et al. (2021) …] are not cited in APA format properly; for example, Kumar, S., Choudhary, S., Singh, G., et al. (2021) are to be cited as Kumar et al. (2021) or, if it is Sahoo, S., and Kumar, S. (2021), to be cited as Sahoo and Kumar (2021).

**Research Methods:**

 It is unclear how the study identifies the heterogeneity among different investor groups and how the strata are formed to choose the sample investors randomly. It requires justification and details on the process of sampling.

 Analysis that relies on respondents’ perceptions collected through the online survey requires cross-validation by collecting some data by visiting to the respondents in person by field survey. So that the study may establish the results' validation and authenticity.

 The study only tested one and only one hypothesis related to psychological biases. It should address the issue of missing out of social biases by formulating the second hypothesis. 

 Table 1 and Table 2 should present the alpha values for each factor of social and psychological biases rather than including all measured variables (33 & 25) at once. I don’t know how the author is presenting social and psychological biases with equal 350 d.f. in the Shapiro-Wilk Test (Table 3). 

 Before moving to confirmatory factor analysis (CFA) using SEM, it is required to extract the only relevant factors of the above two types of biases employing exploratory factor analysis (EFA).

**Results:**

 The study's analysis includes two estimating models that go beyond the hypothesis, raising doubt in the analysis. Again, the first estimating model represents IS, PA, HB, and CBDC as social factors beyond their nature. Furthermore, the second model estimates overconfidence, loss aversion, representativeness, gamblers fallacy, and regret aversion as psychological factors that were not separately identified in the alpha test or derived by EFA.

**Conclusion:**

 The result sounds good, but it lacks discussion on the inherent limitations and delimitations of the study so as to bring robustness to the findings and illustrate the future scope of the study.

 The conclusion drawn primarily seems biased by discussing only the favored variables and not supported by the findings. The findings of the study show statistical significance to all the study variables.

Is the work clearly and accurately presented and does it cite the current literature?

Partly

If applicable, is the statistical analysis and its interpretation appropriate?

No

Are all the source data underlying the results available to ensure full reproducibility?

Partly

Is the study design appropriate and is the work technically sound?

No

Are the conclusions drawn adequately supported by the results?

No

Are sufficient details of methods and analysis provided to allow replication by others?

Partly

Reviewer Expertise:

My area of expertise is finance, with a special emphasis on stock market research, investing methods, and financial economics, particularly behavioral finance.

I confirm that I have read this submission and believe that I have an appropriate level of expertise to state that I do not consider it to be of an acceptable scientific standard, for reasons outlined above.

# Response

**Published:** 2024-12-27

**Authors:** FARMAN ALI

I will be incorporating all the suggestions.Once I get the access to modify it.
